# Effect of HUFA in Enriched *Artemia* on Growth Performance, Biochemical and Fatty Acid Content, and Hepatopancreatic Features of *Penaeus vannamei* Postlarvae from a Commercial Shrimp Hatchery in Santa Elena, Ecuador

**DOI:** 10.1155/2023/7343070

**Published:** 2023-03-28

**Authors:** Marina Martínez Soler, Gercende Courtois de Vicose, Javier Roo Filgueira, José Zambrano Sánchez, Edwin Yugcha Oñate, Magaly Montachana Chimborazo, Walter Intriago Díaz, Eduardo Reyes Abad, Juan Manuel Afonso López

**Affiliations:** ^1^Aquaculture Research Group (GIA), Institute of Sustainable Aquaculture and Marine Ecosystems (IU-ECOAQUA), Universidad de Las Palmas de Gran Canaria (ULPGC), Carretera de Taliarte, s/n, 35214 Telde, Spain; ^2^BIOGEMAR S.A. Company/PRODUMAR Company (ALMAR group), Ciud. Miramar vía Mar Bravo, 240206 Salinas, Santa Elena, Ecuador

## Abstract

A 12-day experiment was conducted to investigate the effects of *Artemia* enrichment with two experimental microalgal emulsions (formulated with selected fatty acid contents) on *Penaeus vannamei* postlarvae. For this purpose, 405,000 postlarvae (stage 1) were obtained from a commercial hatchery in Santa Elena, Ecuador, and distributed into nine fiberglass tanks. Postlarvae were fed for 12 days with three experimental diets (three tanks per treatment): treatment A (*Artemia* enriched with experimental microalgal emulsion A and dry diet), treatment B (*Artemia* enriched with experimental microalgal emulsion B and dry diet) and nonenriched *Artemia* (*Artemia* without enrichment and dry diet). At the end of the experiment, length (mm), coefficient of variation of population sizes, number of postlarvae in a gram of weight (PL-gram), biochemical composition, fatty acid profile, hepatopancreas perimeter, and histopathological hepatopancreas status of *P. vannamei* postlarvae (stage 12) were analyzed. To evaluate the status of the hepatopancreas, a categorization range (1–5) was created with different histological parameters such as number of B cells, vesicles around them, healthy tubules, and degradation tissues. Growth traits did not present differences between treatments; total length was 10.17 mm, 10.83 mm, and 10.27 mm for treatment A, treatment B, and nonenriched treatment, respectively, and PL-gram was 141.00, 162.00, and 142.33 for treatment A, treatment B, and nonenriched treatment, respectively. Biochemical composition of postlarvae (lipids, ash, and protein content) did not present differences between the three treatments. Significant differences were observed in the content of three essential fatty acids (DHA, DPA, and ARA) in *Penaeus vannamei* postlarvae fed with *Artemia* enriched with experimental emulsions. Thus, DHA content was significantly superior in animals fed with *Artemia* enriched with treatments A and B (9.80 ± 0.71% and 9.75 ± 0.44%, respectively) than in animals fed with unenriched *Artemia* (5.78 ± 0.68) (*P* < 0.05). Concerning arachidonic acid (ARA), treatments A and B showed 3.31 ± 0.20% and 3.19 ± 0.09%, respectively, higher than postlarvae fed with unenriched *Artemia*, 2.73 ± 0.04% (*P* < 0.05). Regarding DPA content, treatments A and B reported higher values of MA and MB (0.81 ± 0.06% and 0.86 ± 0.08%, respectively), than unenriched *Artemia* (0.43 ± 0.02%) (*P* < 0.05). Interestingly, the increase in DHA, DPA, and ARA contents in postlarvae coincided with the increase in hepatopancreas perimeter. In addition, a large number of B cells, a large number of healthy tubules, increased dilatation of the central tube, and a lower percentage of deteriorated tissue were observed in the hepatopancreas when postlarvae were fed with enriched *Artemia*.

## 1. Introduction

The world production of white shrimp (*Penaeus vannamei*) has grown from 2.7 million tons in 2010 to 5.8 million tons in 2020 with a value of 26,929 million euros in the first sale [1], being the main global species in relation to production value, ahead of the Atlantic salmon *Salmo salar* [2]. Ecuador is the largest producer in the world with more than 1.2 million tons of shrimp produced in 2022 [3]. This industry has a significant impact on the Ecuadorian economy, being a source of employment and stability for different entities, both public and private [4]. The demand for Ecuadorian postlarvae continued to increase in recent years due to the high development rate of shrimp farms, with increasing demand for high-quality postlarvae; thus, technological investment is focused, to a large extent, on improving the quality of the postlarvae produced.

Postlarvae quality is one of the most important factors in hatcheries, affecting the entire process of growing farmed shrimp [5]. Some of the standard quality indicators of larvae include growth rate and size, nutritional status, general condition, biochemical composition of the body, and hepatopancreas status [5, 6]. Besides, hepatopancreas status can be one of the indicators to determine the shrimp's health status [5, 7]. In this sense, characteristics such as size, large number of well-developed B cells, large number of vesicles around them, dilated tubule, and reduction in degradation tissue are due to an increase in hepatopancreas secretions and indicate improvement in hepatopancreas health [5].

During the early postlarvae (PL) stages, feeding with live prey is still necessary as it provides high digestibility and water quality stability [8] and stimulates digestive enzymes [9–11]. From the beginning of the development of world shrimp aquaculture to date, *Artemia* has been the main live prey supplied to PL [12] because of its size, its great acceptance by PL, and easy storage in the form of cysts [13]. Moreover, Gamboa-Delgado and Le Vay [14] demonstrated that shrimp early postlarvae incorporated higher amounts of nutrients from *Artemia* than from an inert diet, indicating that lower than expected carbon contributions from inert diets to tissue growth may be due to poor digestibility. Proteins are the most abundant component in their natural diet, so it is important to feed PL with live foods rich in this nutrient, such as *Rotifers* and *Artemia*.

Despite the great importance of *Artemia* as live food in PL culture, it lacks some essential nutrients necessary for correct shrimp development and growth [12, 15]. More specifically, a deficiency in essential lipids for PL was reported [12, 16], in particular long-chain polyunsaturated fatty acids (LC-PUFA) such as arachidonic acid (ARA), eicosapentaenoic acid (EPA), and docosahexaenoic acid (DHA) [17–19].

One of the biggest challenges for the Ecuadorian white shrimp industry is to produce high-quality PL, with high growth and production potential. PL with a high content of unsaturated fatty acids (HUFA) and phospholipids, which improve resistance to stress and diseases, have been identified as those with the best quality [20]. In this way, the enrichment of *Artemia* has a fundamental role in the aquaculture shrimp industry for the nutritional improvement of the species [12], where, once enriched with HUFA-rich particles, *Artemia* contains the necessary nutrients for fish and marine crustacean larvae to improve growth, survival, and metamorphosis success [12, 21–23].

Several authors have documented that *Artemia* enrichment with HUFA in *Penaeus* spp. improves the quality of postlarvae and their survival when exposed to stress conditions such as high salinity [20, 24–27].

Limited information is available at the histological level about the effects of HUFA on the hepatopancreatic status of PL shrimp. Therefore, this study was aimed at investigating, during a 12-day trial, the effects of *Artemia* enrichment with microalgal emulsions enriched with fatty acids on growth performance, biochemical profiles, fatty acid profiles, hepatopancreatic perimeter, and hepatopancreatic histological structure of a population of *Penaeus vannamei* postlarvae bred in an Ecuadorian commercial farm.

## 2. Materials and Methods

### 2.1. Postlarvae Rearing

405,000 stage 1 postlarvae (PL1) were obtained from an industrial rearing pond in a commercial hatchery (BIOGEMAR S.A., Santa Elena, Ecuador). All postlarvae were randomly distributed into nine fiberglass tanks (0.5m^3^) at a density of 45,000 PL1 per tank. During the experiment, the environmental conditions were monitored: temperature—28.5 ± 0.5°C, salinity—20 ± 0.3 ppt, and dissolved oxygen—>5 ppm. The microalgae *Thalassiosira* sp. and *Tetraselmis* sp. were added upon storage of PL1 at a density of 3 × 10^4^ cells/mL. Microalgal density was monitored daily and replaced when necessary to maintain initial density. About 50% of the total water was exchanged every day to maintain water quality. Postlarvae were reared during the 12 days of the trial until reaching postlarvae stage 12 (PL12), corresponding to the stage at which they are sold for grow-out in industrial hatcheries. PL12 samples were collected from each experimental tank to analyze growth parameters, biochemical composition (10 grams of biomass per tank), fatty acid composition (10 grams of biomass per tank), hepatopancreas histology (7 PL per tank), and hepatopancreas perimeter (5 PL per tank). *XpertCount™* equipment from *XpertSea™* (Quebec, QC, Canada) was used to estimate length (mm), coefficient of variation of population sizes, and number of postlarvae in a gram of experimental postlarvae (PL-gram).

### 2.2. Artemia Enrichment and Feeding Regime

Two experimental microalgal emulsions were formulated with selected fatty acid content (Table 1) and stored at 4°C until used for *Artemia* enrichment experiments performed at BIOGEMAR S.A., Santa Elena, Ecuador. *Artemia* (high-quality Artemia cysts, *PRIRODA GREEN™*, Guayaquil, Ecuador) were enriched for 18 h in 25 L tanks at a density of 250,000 individuals L^−1^, after opening their mouth (6 hours posthatching). During enrichment, seawater temperature was 28°C, and 0.4 g L^−1^ of each experimental emulsion was added to the tanks under continuous aeration and oxygen supply. During the experiment, subsamples of newly hatched *Artemia* and enriched *Artemia* were collected and analyzed for proximate and fatty acid composition (Table 2).

White shrimp postlarvae were fed every two hours with the three respective experimental diets (triplicate treatments). To imitate the feeding regimes of the company, the postlarvae were alternatively fed the commonly used inert dry diet every two hours between the enriched or unenriched *Artemia* feedings. Therefore, the three experimental diets were as follows: inert dry diet+*Artemia* enriched with microalgae A (TA), inert dry diet+*Artemia* enriched with microalgae B (TB), and inert dry diet+nonenriched *Artemia* (NE). Each feeding was given *Ad libitum* to replicate the feeding methods used under industrial conditions. From PL1 to PL12, postlarvae were fed an increasing quantity of inert dry diet (2 to 4 grams per tank) and an increasing number of *Artemia* per PL (12 *Artemia* per PL to 20 *Artemia* per PL).

### 2.3. Biochemical Analysis

All samples were lyophilized using the Telstar Freeze Dryer model CRYODOS -80 prior to biochemical analysis. Total lipids were extracted and weighed according to the methodology of Folch et al. [28]. Proteins were determined by the Kjeldahl method [29], which is based on total nitrogen composition, and ash content was determined according to the methods described in the AOAC [29]. Fatty acids were extracted by transesterification in sulfuric acid (1%) and methanol [30]. Fatty acids were diluted in hexane, and separation, identification, and quantification were carried out via gas chromatography (GC-14A, Shimadzu, Japan) as described in Izquierdo et al. [31].

### 2.4. Hepatopancreas Status

While the experiment was being carried out, the hepatopancreas status of PL was assessed daily by preparing a wet slide of postlarval specimens to perform microscopic analysis at a magnification of ×10. Healthy PL showed a full hepatopancreas of dark color.

Tissue sections from PL12 were used to examine anatomical abnormalities in the hepatopancreas. For this purpose, seven PL were collected from each tank (21 per treatment) and transferred to containers containing Davidson's solution. After fixation, they were processed and embedded in paraffin blocks according to routine histological procedures and sectioned at 0.5 *μ*m–0.7 *μ*m using a microtome (*Leica Reichert Jung AUTOCUT 2055*). The Hematoxylin and Eosin (H&E) stained tissue sections were observed via light microscopy and analyzed using a computerized image analyzer (Image-Pro Plus software). Hepatopancreas perimeter was measured by Image-Pro Plus software (magnification of ×4). To determine the hepatopancreas status (development, health and quality) in *P. vannamei* PL12, various parameters were recorded: quantity of B cells, quantity of vesicles around them, number of healthy tubules, dilated central tube, and percent of tissue degeneration [5, 7, 32], which were then used to design a categorization range for hepatopancreas quality (1-5) (magnification of ×10) (Table 3).

### 2.5. Data Analysis

One-way ANOVA tests were performed using the R statistical program. Variations in growth, biochemical analysis, fatty acid profile, hepatopancreas perimeter, and hepatopancreas categorization were studied. Normality was checked using the Kolmogorov-Smirnoff test and the homogeneity of variances with the Bartlett test. The Tukey test (HSD) was carried out to execute the test a posteriori. Data were presented as mean ± standard deviation (SD).

## 3. Results

### 3.1. Artemia Enrichment

Lipid and fatty acid profiles (TFA%) of *Artemia* enriched with both experimental emulsions did not present significant differences between treatments (TA and TB) (TFA%) (Table 2). *Artemia* fed with microalgae A (MA) presented 19.8% of lipids; *Artemia* fed with microalgae B (MB) showed 17.76% of lipids; and unenriched *Artemia* showed 17.3% of lipids (Table 2). The DHA content in enriched *Artemia* increased from 0.61 to 3.15% TFA compared with unenriched *Artemia* (Table 2). The DPA content in enriched *Artemia* increased from 0.23 to 0.65% compared with unenriched *Artemia* (Table 2). The ARA and EPA content was very similar in the three treatments.

### 3.2. Growth Performance

At the end of the trial, *P. vannamei* postlarvae mean total length, coefficient of variation of population sizes and number of postlarvae in a gram of weight (PL-gram) did not present significant differences between the three treatments (Table 4).

### 3.3. Biochemical Composition and Fatty Acid Profile

Postlarvae total lipid, ash and protein content did not show significant differences between TA, TB, and control diet (postlarvae fed with non-enriched *Artemia*) (Table 5).

In terms of fatty acid profile, postlarvae DHA content was significantly superior in animals fed with *Artemia* enriched with MA and MB (9.80 ± 0.71% and 9.75 ± 0.44%, respectively) than those fed with unenriched *Artemia* (5.78 ± 0.68) (*P* < 0.05) (Table 5). Consequently, DHA/EPA and DHA/ARA indexes were superior in postlarvae fed with enriched *Artemia* (TA and TB) (Table 5). Postlarvae (PL12) fed with *Artemia* enriched with MA and MB showed a higher concentration of arachidonic acid (ARA) (3.31 ± 0.20%and 3.19 ± 0.09%, respectively), than postlarvae fed with unenriched *Artemia* (2.73 ± 0.04%) (*P* < 0.05) (Table 5). Postlarval DPA content was significantly superior in the treatment with *Artemia* enriched with MA (0.81 ± 0.06%) and MB (0.86 ± 0.08%) in comparison with that observed in animals fed with unenriched *Artemia* (0.43 ± 0.02%) (*P* < 0.05). Nevertheless, postlarval EPA content did not present significant differences between treatments (*P* > 0.05) (Table 5).

### 3.4. Hepatopancreas Status

The hepatopancreas perimeter was significantly higher in postlarvae fed with enriched *Artemia* (TA and TB: 1960.13 *μ*m ± 262.80 *μ*m and 1934.87 *μ*m ± 294.20 *μ*m, respectively) than in postlarvae fed with unenriched *Artemia* (1664.93 *μ*m ± 328.10 *μ*m) (*P* < 0.05). The score between treatments for hepatopancreas status categorization (Table 3) was higher in postlarvae fed with enriched *Artemia* (TA and TB: 3.38 ± 0.92 and 3.33 ± 0.58, respectively) than those fed with unenriched *Artemia* (2.91 ± 0.77), although no significant differences were found (*P* > 0.05).

**Figure 1:**
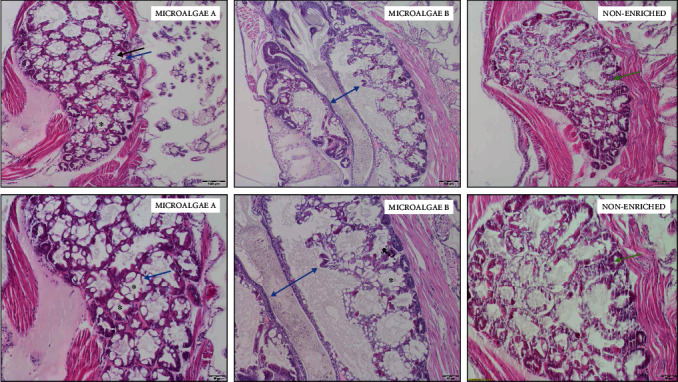
Photomicrograph of a longitudinal section of hepatopancreas from postlarvae fed with different enriched and unenriched *Artemia*. Microalgae A: large number of B cells (blue arrow), well-developed tubules (black arrow), and clearly dilated tubules (asterisks). Microalgae B: increased number of vesicles around B cells are obvious, healthy, and dilated tubules (asterisks). The central tube was also dilated (double blue arrow). Nonenriched: fewer B cells and presence of degenerated tissue (green arrow).

According to the microscopic study of *P. vannamei* postlarvae, the hepatopancreas of PL in TA was apparently healthy and well structured. The hepatopancreatic tissue presented a large number of well-developed B cells; no degeneration of the tubule's lumen was observed. Moreover, the central tube was dilated (Figure 1(a)). Hepatopancreatic tissue of postlarvae from TB is shown in Figure 1(b). The hepatopancreas is well developed, presenting many vesicles and B cells surrounding healthy tubules and a slight increase in lipid deposition in comparison with PL tissues from TA. The hepatopancreas of *P. vannamei* postlarvae fed with *Artemia* without enrichment presented a large portion of degenerated tissues, mostly in layers surrounding the organ, as well as a lower number of B cells and few healthy tubules and vesicles (Figure 1(c)). Although there were no significant differences between treatments in terms of the categorization score, there was an obvious distinction between treatments as regards the presence of B cells, vesicles, healthy and well-developed tubules, and degenerated tissue.

## 4. Discussion

### 4.1. Nutritional Value of Artemia

In this study, the proximate composition (%) and fatty acid profile of enriched *Artemia* reflected the values of the experimental emulsions (MA and MB) used in the *Artemia* enrichment process, especially in the content of essential fatty acids such as DHA and DPA. Generally, the fatty acid profile of *Artemia* enriched with experimental emulsions was similar to previously reported profiles obtained with commercial products such as Olio w-3®, Red pepper ®, Top Rich®, Culture Selco®, microalgae mix of *Dunaliella salina*, and *Chlorella vulgaris* [9] and experimentally prepared n-3 HUFA emulsions [9, 33]. Both experimental emulsions presented a similar fatty acid profile; therefore, no significant differences were detected in the profile of *Artemia* enriched for 18 h with each product.

The percentage of DHA in unenriched *Artemia* nauplii was 0.61%, although some authors reported lower percentages, e.g., 0.08% [33]. After enrichment with MA and MB, the DHA concentration in *Artemia* increased up to 3.25%.

### 4.2. Growth Performance

Other longer-term studies found significant differences in *Penaeus* spp. postlarval growth parameters when fed with enriched *Artemia* [20, 34]. In the present experiment, no differences were observed in growth parameters (length, PL-gram, and coefficient of variation of population sizes) perhaps due to the short period of postlarvae culture (12 days). Putra et al. [35] reported that 12-day *Artemia* enrichment with gamma emulsions (EPA and DHA) had no significant effect on the specific growth of *P. vannamei*.

### 4.3. Fatty Acid Profile

No information has been reported about the effect of enriched *Artemia* on the fatty acid profile of *P. vannamei* PL after just 12 days of experimentation, corresponding to the PL production time of commercial hatcheries. During this experiment, PL quality improved significantly in terms of essential fatty acid contents (DHA, DPA, and ARA) when postlarvae were fed with enriched *Artemia* (TA and TB).

In the present study, DHA levels in both experimental emulsions (MA and MB) were elevated and showed a significant effect on the content of this fatty acid in PL fed with enriched *Artemia* compared with unenriched *Artemia.* Similarly, several previous reports supported that the DHA content in *P. vannamei* PL was higher when they were fed with *Artemia* enriched with commercial products such as Easy-DHA Selco after 15 days of experimentation (INVE Aquaculture, Dendermonde, Belgium) [34, 36]. The DHA content in PL fed with enriched *Artemia* (TA and TB) was 1.7 times higher than that of PL fed unenriched *Artemia* (NE). These results were very similar to those obtained by Ahmadi et al. [34], who reported a DHA content in *P. vannamei* PL fed with *Artemia* enriched with commercial supplements 2.5 times higher than that in PL fed unenriched *Artemia.* Generally, the postlarvae fatty acid profile reported in this study was similar to reported by Ahmadi *et al*. [34]. No significant differences were found in PL for EPA content, and according to Ahmadi et al. [34], the EPA content in *P. vannamei* PL was higher when fed with unenriched *Artemia* than with enriched *Artemia*. On this matter, some authors reported that the EPA content in the muscle of marine species is not affected when the food integrates microalgal compounds [37–39]. Highly unsaturated fatty acids (HUFA) such as EPA and DHA are important components of phospholipids in cell membranes and affect membrane fluidity, lipid development and metabolism, reproductive development, and various functions of the cell immune system in marine species [40–45].

The results from this study indicated that ARA levels were significantly higher in PL fed with enriched *Artemia*, although this fatty acid was not present in large quantities in the experimental emulsions. Eryalcin [9] reported that when rotifers were enriched with microalgae mix, ARA had significantly higher levels than those rotifers enriched with commercial products (Olio w-3®, Red pepper ®, Top Rich®, Culture Selco®). ARA is not considered essential in species such as *P. japonicus* [43], but it is important for the immune system and eicosanoid synthesis, being physiologically active in most aquatic organisms [46, 47]. The ARA content in *Artemia* was associated with an improvement in the growth of *P. vannamei* [48], being more effective than other PUFAs but less essential than DHA and EPA [49]. The increase in ARA content in PL significantly decreased the EPA/ARA index with respect to unenriched *Artemia.* It is important to control this index (EPA/ARA) since both fatty acids have a similar structure and compete enzymatically in eicosanoid synthesis [46, 47, 50, 51]. Therefore, appropriate ARA requirements should be defined for *P. vannamei* PL in future research.

Furthermore, DHA and DPA are deposited in the cellular membranes, in *Penaeus monodon* larvae, when fed with supplemented microalgae [52]. In marine fish larvae, DPA is positively correlated with growth and survival [53, 54]. However, the knowledge of DPA effects is even scarce in comparison with other LC-PUFA, like DHA. So, DPA opens new perspectives to understand its role in marine larvae development.

### 4.4. Hepatopancreas Status

The quality of the early postlarvae stages in shrimp is difficult to evaluate using only parameters such as weight gain and survival; therefore, microscopic criteria need to be evaluated [32]. In this respect, the hepatopancreas is one of the most important organs in shrimp, synthesizing, transporting, and secreting digestive enzymes and storing lipids, glycogen, and minerals [32, 55] and where most enzymes are produced [56]. Characteristics such as tubule formation, color (dark or pale), and hepatopancreas size can be used as indicators of nutritional quality in shrimp [32, 57]. In the present study, the hepatopancreas status of *P. vannamei* PL wet samples was observed daily under light microscopy. The brown coloration observed of the hepatopancreas was an indication of good health parameters [7].

As this organ is very sensitive to different diets [7, 58], shrinkage in size easily indicates negative effects [7]. At the end of the trial, the hepatopancreas perimeter was significantly higher in PL fed with enriched *Artemia* than with unenriched *Artemia.* Therefore, it appears that feeding live prey enriched with HUFA to postlarvae was beneficial for PL health and was reflected in hepatopancreas size.

Little information on the histological effects of HUFA in the hepatopancreas of *P. vannamei* PL during the early stages has been reported, even though it is one of the indicators of the shrimp's health status [7, 59]. In the present study, the hepatopancreas of PL fed with enriched *Artemia* with both experimental emulsions (TA and TB) seemed healthy and well structured, with a large number of well-developed B cells, dilated tubule, and a reduction in degradation tissue. These latter observations were due to an increase in hepatopancreas secretions [5] and coincided with a higher content of unsaturated fatty acids such as DHA, DPA, and ARA. The hepatopancreas of *P. vannamei* PL fed with unenriched *Artemia* presented a large portion of degenerated tissue surrounding the organ and a lower number of B cells.

B cells are most abundant in hepatopancreas tissue, highly vacuolated, and involved in intracellular digestion and nutrient absorption [60–62]. Moh et al. [60] reported an increase in B cell number when supplementation with *Morinda citrifolia* fruit was incorporated into *P. vannamei* diets, which potentially improved the conversion of F cells to B cells, signifying higher intracellular digestion and nutrient absorption. However, Moh et al. [60] did not report the PL fatty acid profile to establish a congruence between both quality criteria. Araújo et al. [63] reported a decreasing number of B and R cells in the hepatopancreas of *P. vannamei* juveniles (3.0 g) when ARA was included in the diet, probably due to an alteration in gene expression related to eicosanoid synthesis because of a decrease in DHA and EPA content in the organ. In the present study, the DHA content of PL fed with enriched *Artemia* was three times higher than the ARA content; as a result, the DHA/ARA index was significantly higher.

A HUFA deficiency can cause more lipid vacuoles and incomplete cells in the hepatopancreas of *P. vannamei* early-stage juveniles, but an excess could cause damage [40]. Damage was not observed in the present study, signifying that the HUFA content in both experimental emulsions used to enrich *Artemia* diets was well adapted to postlarval requirements. These results highlighted the need for future studies to establish the specific influence of fatty acid composition on hepatopancreatic cell morphology and status in shrimp. It is important to determine the precise amount of HUFA that does not cause oxidative damage to the hepatopancreas since, according to An et al. [40], the content of MDA (malondialdehyde) in this organ, which indicates the degree of oxygen free radical damage in cells [40, 64], increased with increasing dietary HUFA levels.

In conclusion, twelve days of culture is sufficient to significantly increase the content of unsaturated fatty acids, such as DHA, DPA, and ARA, in *Penaeus vannamei* postlarvae by enriching *Artemia* with formulated microalgal emulsions, allowing us to obtain higher-quality postlarvae. In addition, HUFA enrichment improves the hepatopancreas status and health of postlarvae with respect to size, number of B cells and vesicles, quantity of healthy tubules, dilatation of central tube, and surface of degenerated tissue.

## Figures and Tables

**Table 1:** Fatty acid composition (%TFA) of two experimental emulsions with selected fatty acid contents (percentage of total fatty acids (%TFA)).

Fatty acid content (%TFA)	Microalgae A	Microalgae B
n-3	43.83	42.92
n-6	16.61	17.78
Total, n-3 HUFA	43.07	42.92
14:0	0.41	0.39
16:0	15.00	14.69
16:1 n-7	0.54	0.66
18:0	1.69	1.75
18:1 n-7	0.46	0.52
18:1 n-9	3.41	3.74
18:1 n-7	0.46	0.52
18:2 n-6	4.62	4.22
18:3 n-3	0.52	0.45
20:1 n-9	0.11	0.10
20:4n-6 (ARA)	2.52	2.48
20:5n-3 (EPA)	3.32	3.32
22:6n-3 (DHA)	37.96	37.22
DHA/EPA	11.40	11.21
EPA/ARA	1.32	1.34

HUFA: highly unsaturated fatty acid; ARA: arachidonic acid; DHA: docosahexaenoic acid; EPA: eicosapentaenoic acid.

**Table 2:** Lipid (%dry matter) and fatty acid composition (%TFA) of *Artemia* Nauplii after 18 h enrichment with two experimental emulsions (MA and MB). Fatty acid content data is presented as means ± SD (*n* = 3).

Fatty acid content (%TFA)	*Nonenriched*	Microalgae A	Microalgae B
Lipids	17.30	19.80 ± 0.44	17.76 ± 2.22
n-3	35.56	7.65 ± 2.58	9.77 ± 0.99
n-6	8.14	2.90 ± 0.68	3.74 ± 0.58
Total, n-3 HUFA	3.33	2.63 ± 0.69	5.13 ± 1.52
14:0	1.00	0.55 ± 0.08	0.39 ± 0.36
16:0	13.71	23.03 ± 3.5	23.23 ± 1.74
16:1 n-7	2.23	2.30 ± 0.15	2.35 ± 0.11
18:0	6.26	16.60 ± 0.66	16.33 ± 0.76
18:1 n-7	6.36	10.17 ± 0.77	9.74 ± 0.70
18:1 n-9	20.66	27.54 ± 1.5	25.76 ± 1.29
18:2 n-6	6.35	1.81 ± 0.47	2.03 ± 0.29
18:3 n-3	27.93	4.24 ± 1.8	3.91 ± 0.62
20:1 n-9	0.02	2.46 ± 0.13	2.19 ± 0.41
20:4n-6 (ARA)	0.49	0.39 ± 0.08	0.54 ± 0.12
20:5n-3 (EPA)	0.76	0.86 ± 0.13	1.20 ± 0.34
22:6n-3 (DHA)	0.61	1.16 ± 0.43	3.15 ± 1.47
22:5n-6 (DPA)	0.23	0.27 ± 0.05	0.65 ± 0.29
22:5n-3	0.01	0.11 ± 0.05	0.07 ± 0.04
DHA/EPA	0.81	1.34 ± 0.37	2.87 ± 1.70
EPA/ARA	1.56	2.21 ± 0.12	2.23 ± 0.37
DHA/DPA	2.68	1.34 ± 0.37	2.87 ± 1.70

HUFA: highly unsaturated fatty acid; ARA: arachidonic acid; DHA: docosahexaenoic; EPA: eicosapentaenoic acid.

**Table 3:** Categorization range created to measure the hepatopancreas status of *P. vannamei* postlarval stage 12.

Score	B cells	Vesicles	Healthy tubules	Degradation tissue
5	>100	<50	>40	0
4	70–100	>50	20–40	5%
3	50–70	>50	10	10%
2	20–50	>50	5	25%
1	<10	>50	0	>30%

**Table 4:** Total length (mm), coefficient of variation of population sizes (%), and number of postlarvae in a gram weight of *P. vannamei* (PL12) (mean ± SD, *n* = 3).

Parameters	Nonenriched	Treatment A	Treatment B
Total, length (mm)	10.27 ± 0.52	10.17 ± 0.50	10.83 ± 0.32
CV%	17.51 ± 0.54	19.47 ± 2.25	17.53 ± 1.65
PL-gram	142.33 ± 34.2	141.00 ± 8.00	162.00 ± 9.56

**Table 5:** Proximate (lipid, ash, and protein content (%dry matter)) and fatty acid composition (%TFA) of *P. vannamei* postlarvae 1 (PL1) and 12 (PL12) fed enriched *Artemia* with different experimental emulsions and unenriched *Artemia*. Lipid contents and FA data represent means ± SD (*n* = 3). Different superscripts within each row indicate a significant difference between diets (ANOVA (*P* ≤ 0.05), Tukey's HSD).

	PL1	PL12–treatment A	PL12–treatment B	PL12–nonenriched
Lipids	10.81	9.97 ± 0.96	10.83 ± 0.63	9.42 ± 0.57
Ash	22.50	20.86 ± 0.61	20.70 ± 0.61	20.30 ± 0.61
Protein	59.98	65.00 ± 0.76	63.25 ± 0.72	64.93 ± 3.63
Fatty acid content (%FTA)				
n-3	29.25	31.09 ± 0.79^a^	30.58 ± 0.99^ab^	28.47 ± 1.23^b^
n-6	10.26	12.82 ± 0.28	13.13 ± 0.28	12.07 ± 0.42
14:0	0.64	0.22 ± 0.02	0.22 ± 0.04	0.30 ± 0.03
16:0	14.76	12.38 ± 0.70	11.70 ± 1.00	12.65 ± 0.38
16:1 n-7	3.36	1.87 ± 0.14	1.92 ± 0.14	2.20 ± 0.20
18:0	9.02	8.24 ± 0.08	8.07 ± 0.22	8.33 ± 0.27
18:1 n-7	5.95	7.36 ± 0.07	7.30 ± 0.26	7.70 ± 0.23
18:1 n-9	18.01	18.11 ± 1.04	18.57 ± 0.75	19.84 ± 0.31
18:2 n-6	6.61	7.38 ± 0.37	7.80 ± 0.44	7.75 ± 0.44
18:3 n-3	6.48	6.28 ± 0.56	6.35 ± 0.29	6.84 ± 0.31
20:1 n-9	0.03	1.35 ± 0.05	1.38 ± 0.03	1.41 ± 0.08
20:4n-6 (ARA)	2.02	3.31 ± 0.20^a^	3.19 ± 0.09^a^	2.73 ± 0.04^b^
20:5n-3 (EPA)	14.00	13.04 ± 0.71	12.46 ± 0.50	13.71 ± 0.59
22:6n-3 (DHA)	6.67	9.80 ± 0.71^a^	9.75 ± 0.44^a^	5.78 ± 0.67^b^
22:5n-6 (DPA)	0.39	0.81 ± 0.06^a^	0.86 ± 0.07^a^	0.43 ± 0.02^b^
22 : 5 n-3	0.39	0.63 ± 0.02^a^	0.65 ± 0.11^a^	0.61 ± 0.10^b^
DHA/EPA	0.48	0.75 ± 0.02^a^	0.78 ± 0.03^a^	0.42 ± 0.03^b^
EPA/ARA	6.94	3.94 ± 0.03^a^	3.91 ± 0.05^a^	5.01 ± 0.18^b^
DHA/DPA	16.98	12.12 ± 1.56	11.21 ± 0.69	13.32 ± 1.25
DHA/ARA	3.31	2.96 ± 0.04^a^	3.06 ± 0.12^a^	2.11 ± 0.23^b^
ARA/EPA	0.14	0.25 ± 0.00^a^	0.26 ± 0.00^a^	0.20 ± 0.01^b^
Total, n-3 HUFA	22.08	24.32 ± 1.33^a^	23.73 ± 0.93^ab^	21.09 ± 1.43^b^
n-3/n-6	2.85	2.43 ± 0.11	2.33 ± 0.12	2.36 ± 0.17

## Data Availability

All the data can be obtained in the manuscript and supplementary materials.

## Additional Points

*Author Statement*. Juan Manuel Afonso López, corresponding author, declares that the information reported in this paper is original, where new formulated diets have been tested to enrich *Artemia* used for feeding postlarvae of shrimp and evaluating their effects on growth and health of postlarvae.

## Supplementary Materials

Supplementary Materials Fatty acid profiles of Artemia enriched by each experimental emulsions, two experimental emulsions, and postlarvae fed with three experimental treatments. Parameters studied considering their sampling factors, replicate, etc. Figures and tables that are included in this manuscript separately.
