# Supplementary material for: Effect of HUFA in Enriched Artemia on Growth Performance, Biochemical and Fatty Acid Content, and Hepatopancreatic Features of Penaeus vannamei Postlarvae from a Commercial Shrimp Hatchery in Santa Elena, Ecuador
Source: Aquac Nutr. 2023 Mar 28;2023:7343070. doi: 10.1155/2023/7343070 (PMC10072957; doi:10.1155/2023/7343070)
Supplement: Supplementary Materials — Fatty acid profiles of Artemia enriched by each experimental emulsions, two experimental emulsions, and postlarvae fed with three experimental treatments. Parameters studied considering their sampling factors, replicate, etc. Figures and tables that are included in this manuscript separately. [file 7343070.f1.zip › Artemia_Emulsion_Postlarvae Fatty Acid composition2.pdf]

Fatty acid profile experimetal emuslions MA & MB

Ácidos grasos	14:00	14:1n-5	14:1n-7	15:00	15:1n-5	16:00	16:1 n-7	16:1n-5	16:2n-4	17:00
**PA**	0,41	0,18	0,00	0,73	0,03	**15,00**	0,54	0,04	0,02	0,01
**PB**	0,39	0,18	0,00	0,69	0,01	**14,69**	0,66	0,02	0,05	0,04
**Ácidos grasos**	**16:3n-1**	**16:4n-3**	**16:4 n-1**	**18:00**	**18:1 n-9**	**18:1 n-7**	**18:1 n-5**	**18:2n-9**	**18:2 n-6**	**18:2n-4**
**PA**	0,12	0,03	0,01	1,69	3,41	0,46	0,02	0,01	4,62	0,03
**PB**	0,02	0,02	0,01	1,75	3,74	0,52	0,02	0,01	6,22	0,03
**Ácidos grasos**	**18: 3n-6**	**18: 4 n-6**	**18:3n-4**	**18:3 n-3**	**18:3n-1**	**18:4 n-3**	**18:4 n-1**	**20:00**	**20:1 n-9**	**20: 1n-7**
**PA**	1,56	0,00	0,05	0,52	0,00	0,20	0,01	0,32	0,11	0,69
**PB**	1,40	0,00	0,03	0,45	0,00	0,15	0,01	0,32	0,10	0,66
**Ácidos grasos**	**20: 1n-5**	**20: 2n-9**	**20:2 n-6**	**20:3n-9**	**20:3 n-6**	**20:4 n-6**	**20: 3n-3**	**20:4 n-3**	**20:5 n-6**	**20:5 n-3**
**PA**	0,09	0,03	0,19	0,01	0,34	2,52	0,11	0,50	0,00	3,32
**PB**	0,09	0,03	0,18	0,01	0,33	2,48	0,11	0,49	0,00	3,32
**Ácidos grasos**	**22:00**	**22:1 n-11**	**22:1 n-9**	**22: 1 n-7**	**22:3 n-6**	**22:4 n-6**	**22:5 n-6**	**22:4n-3**	**22:5 n-3**	**22:6 n-3**
**PA**	0,00	0,22	0,24	0,00	0,00	0,21	7,18	0,00	1,17	**37,96**
**PB**	0,00	0,21	0,25	0,00	0,00	0,20	6,96	0,00	1,12	**37,22**
