# Supplementary material for: Effect of HUFA in Enriched Artemia on Growth Performance, Biochemical and Fatty Acid Content, and Hepatopancreatic Features of Penaeus vannamei Postlarvae from a Commercial Shrimp Hatchery in Santa Elena, Ecuador
Source: Aquac Nutr. 2023 Mar 28;2023:7343070. doi: 10.1155/2023/7343070 (PMC10072957; doi:10.1155/2023/7343070)
Supplement: Supplementary Materials — Fatty acid profiles of Artemia enriched by each experimental emulsions, two experimental emulsions, and postlarvae fed with three experimental treatments. Parameters studied considering their sampling factors, replicate, etc. Figures and tables that are included in this manuscript separately. [file 7343070.f1.zip › Artemia_Emulsion_Postlarvae Fatty Acid composition3.pdf]

	Fatty acid profile of postlarvae fed with Artemia treatments								
Fatty acid	T1-PA	T2-PB	T3-SE	T4-PA	T5-PB	T6-SE	T7-PA	T8-PB	T9-SE
**14:00**	0,24	0,19	0,29	0,21	0,21	0,34	0,22	0,27	0,28
14:1n-5	0,20	0,24	0,32	0,26	0,27	0,37	0,23	0,24	0,25
14:1n-7	0,01	0,02	0,01	0,01	0,01	0,01	0,01	0,00	0
**15:00**	0,30	0,26	0,35	0,28	0,31	0,38	0,26	0,31	0,29
**16:0iso**	0,29	0,3	0,32	0,3	0,35	0,34	0,29	0,27	0,27
15:1n-5	0,00	0,01	0,01	0,01	0,01	0,01	0,01	0,01	0,01
**16:00**	13,16	10,59	12,46	11,82	11,95	13,08	12,15	12,55	12,4
16:1 n-7	1,81	1,76	2,18	2,03	1,99	2,41	1,77	2,01	2,01
16:1n-5	0,65	0,68	0,82	0,74	0,76	0,86	0,71	0,65	0,72
16:2n-4	0,02	0,03	0,04	0,04	0,03	0,05	0,03	0,03	0,03
**17:00**	0,05	0,02	0,03	0,02	0,02	0,07	0,03	0,02	0,02
16:3n-4	0,71	0,73	0,81	0,81	0,79	0,84	0,75	0,76	0,78
16:3n-3	0,15	0,19	0,18	0,16	0,2	0,17	0,17	0,16	0,16
16:3n-1	2,34	1,59	2,01	1,79	1,8	1,99	1,95	1,96	2,01
16:4n-3	0,06	0,04	0,07	0,04	0,05	0,05	0,07	0,06	0,06
**18:00**	8,29	7,83	8,06	8,15	8,15	8,33	8,29	8,24	8,59
18:1 n-9	16,91	17,71	19,48	18,76	18,92	20,07	18,67	19,08	19,98
18:1 n-7	7,28	7,17	7,80	7,42	7,60	7,87	7,39	7,13	7,44
18:1 n-5	0,17	0,17	0,18	0,17	0,18	0,19	0,17	0,19	0,18
18:2n-9	0,08	0,10	0,10	0,17	0,10	0,14	0,08	0,09	0,11
**18:2 n-6**	7,05	7,32	7,25	7,31	7,89	7,99	7,77	8,18	8,02
18:2n-4	0,07	0,09	0,08	0,09	0,09	0,07	0,08	0,09	0,07
18:3 n-9	0,00	0,00	0,00	0,00	0,00	0,00	0,00	0,00	0,00
18: 3n-6	0,18	0,20	0,19	0,22	0,21	0,20	0,20	0,19	0,16
18: 4 n-6	0,00	0,00	0,00	0,00	0,00	0,00	0,00	0,00	0,00
18:3n-4	0,06	0,06	0,07	0,07	0,06	0,07	0,06	0,06	0,06
**18:3 n-3**	5,64	6,24	6,84	6,72	6,68	7,15	6,47	6,13	6,53
**18:3n-1**	0,02	0,02	0,02	0,02	0,02	0,02	0,01	0,02	0,02
**18:4 n-3**	0,27	0,25	0,32	0,29	0,27	0,33	0,28	0,27	0,29
18:4 n-1	0,04	0,04	0,05	0,04	0,05	0,05	0,08	0,03	0,04
**20:00**	0,36	0,34	0,36	0,36	0,34	0,39	0,34	0,33	0,36
**20:1 n-9**	1,30	1,39	1,40	1,40	1,34	1,34	1,36	1,41	1,50
20: 1n-7	0,21	0,29	0,29	0,26	0,27	0,23	0,25	0,32	0,34
20: 1n-5	0,31	0,36	0,36	0,36	0,36	0,37	0,34	0,33	0,36
20: 2n-9	0,01	0,02	0,01	0,01	0,01	0,01	0,01	0,01	0,02
20:2 n-6	0,75	0,82	0,70	0,76	0,80	0,68	0,74	0,78	0,73
20:3n-9+n-	0,02	0,01	0,02	0,02	0,02	0,02	0,02	0,01	0,02
20:3 n-6	0,16	0,19	0,16	0,19	0,20	0,16	0,18	0,18	0,16
**20:4 n-6, ARA**	3,54	3,25	2,78	3,21	3,22	2,71	3,18	3,09	2,71
20: 3n-3	0,56	0,64	0,64	0,64	0,63	0,62	0,60	0,60	0,70
20:4 n-3	0,22	0,25	0,25	0,27	0,25	0,23	0,27	0,25	0,28
20:5 n-6	0,00	0,00	0,00	0,00	0,00	0,00	0,00	0,00	0,00
**20:5 n-3, EPA**	13,84	12,84	14,15	12,75	12,65	13,04	12,52	11,90	13,93
**22:00**	0,00	0,00	0,00	0,00	0,00	0,00	0,00	0,00	0,00
22:1 n-11	0,31	0,46	0,43	0,45	0,41	0,34	0,40	0,50	0,59
22:1 n-9	0,07	0,09	0,10	0,09	0,08	0,09	0,08	0,09	0,10
22: 1 n-7	0,00	0,00	0,00	0,00	0,00	0,00	0,00	0,00	0,00
22:3 n-6	0,00	0,00	0,00	0,00	0,00	0,00	0,00	0,00	0,00
22:4 n-6	0,11	0,09	0,08	0,20	0,08	0,07	0,25	0,11	0,17
**22:5 n-6, DPA**	0,77	0,94	0,45	0,88	0,86	0,42	0,78	0,79	0,43
22:4n-3	0,00	0,00	0,25	0,00	0,00	0,00	0,00	0,00	0,00
**22:5 n-3**	0,61	0,74	0,72	0,65	0,68	0,58	0,62	0,52	0,53
**22:6 n-3, DHA**	10,61	10,25	6,20	9,35	9,40	5,00	9,43	9,59	6,13
**Total n-3**	31,97	31,43	29,63	30,86	30,81	27,18	30,43	29,49	28,60
**Total n-6**	12,57	12,81	11,60	12,78	13,27	12,24	13,11	13,31	12,39
**DHA/DPA**	13,72	10,93	13,75	10,60	10,90	11,91	12,03	12,12	14,30
**EPA/ARA**	3,91	3,95	5,09	3,97	3,93	4,81	3,94	3,85	5,14
**ARA/EPA**	0,26	0,25	0,20	0,25	0,25	0,21	0,25	0,26	0,19
**DHA/EPA**	0,77	0,80	0,44	0,73	0,74	0,38	0,75	0,81	0,44
**DHA/ARA**	3,00	3,15	2,23	2,91	2,92	1,85	2,97	3,11	2,26
**Total n-3 HUFA**	25,85	24,71	22,22	23,66	23,61	19,48	23,44	22,87	21,57
**n-3/n-6**	2,54	2,45	2,55	2,42	2,32	2,22	2,32	2,21	2,31
