# Supplementary material for: Effect of HUFA in Enriched Artemia on Growth Performance, Biochemical and Fatty Acid Content, and Hepatopancreatic Features of Penaeus vannamei Postlarvae from a Commercial Shrimp Hatchery in Santa Elena, Ecuador
Source: Aquac Nutr. 2023 Mar 28;2023:7343070. doi: 10.1155/2023/7343070 (PMC10072957; doi:10.1155/2023/7343070)
Supplement: Supplementary Materials — Fatty acid profiles of Artemia enriched by each experimental emulsions, two experimental emulsions, and postlarvae fed with three experimental treatments. Parameters studied considering their sampling factors, replicate, etc. Figures and tables that are included in this manuscript separately. [file 7343070.f1.zip › Artemia_Postlarvae_Hepatopancrean_Hperimeter2.pdf]

Growth parameters, proximate and fatty acid profile of P. vannamei postlavae fed Artemia treatments

enriquecimiento	zona	replica	bm	superv	plg	long	lip	acgrasos	cv	pf	prot	min	dha	aa	dpa	cator	224n6	totaln3	totaln6	dha/dpa	ara/epa	ara/epa	dha/epa	dha/ara	hufa	epa
A	exterior	1	241,2	62	103	10,57	8,86575	0,0133	18,13	24843,6	65,82095	21,5534	10,61	3,54	0,77	0,24	0,11	31,97	12,57	13,72	3,91	0,26	0,77	3	25,85	13,84
A	interior	2	226,3	90	159	9,67	10,6107	0,0149	17,15	35981,7	64,87605	20,3857	9,35	3,21	0,88	0,21	0,2	30,86	12,78	10,6	3,97	0,25	0,73	2,91	23,66	12,75
A	medio	3	204,12	84	165	10,58	10,431	0,0141	17,24	33679,8	64,312	20,641	9,43	3,18	0,78	0,22	0,25	30,43	13,11	12,3	3,94	0,25	0,75	2,97	23,44	12,52
B	exterior	1	178,3	63	141	10,17	11,5465	0,0176	19,465	25140,3	62,61535	20,5346	10,25	3,25	0,94	0,19	0,09	31,43	12,81	10,93	3,95	0,25	0,8	3,15	24,71	12,84
B	interior	2	241,4	90	149	10,67	10,3889	0,018	21,71	35968,6	64,03965	21,3857	9,4	3,22	0,86	0,21	0,08	30,81	13,27	1,9	3,93	0,25	0,74	2,92	23,61	12,65
B	medio	3	115,2	38	133	9,67	10,5485	0,0181	17,22	15321,6	63,0943	20,1754	9,59	3,09	0,79	0,27	0,11	29,49	13,31	12,12	3,85	0,26	0,81	3,11	22,87	11,9
NE	exterior	1	249,8	63	101	11,19	10,06295	0,0124	19,34	25229,8	61,35715	24,4048	6,2	2,78	0,45	0,29	0,08	29,63	11,6	13,75	5,09	0,2	0,44	2,23	22,22	14,15
NE	interior	2	223,4	92	165	10,57	9,23435	0,0029	16,11	36861	64,80385	18,87	5	2,71	0,42	0,34	0,07	27,18	12,24	11,91	4,81	0,21	0,38	1,85	19,48	13,04
NE	medio	3	198,3	109	220	10,74	8,9709	0,013	17,15	43626	68,61895	17,647	6,13	2,71	0,43	0,28	0,17	28,6	12,39	14,3	5,14	0,19	0,44	2,26	21,57	13,93
