# Supplementary material for: Effect of HUFA in Enriched Artemia on Growth Performance, Biochemical and Fatty Acid Content, and Hepatopancreatic Features of Penaeus vannamei Postlarvae from a Commercial Shrimp Hatchery in Santa Elena, Ecuador
Source: Aquac Nutr. 2023 Mar 28;2023:7343070. doi: 10.1155/2023/7343070 (PMC10072957; doi:10.1155/2023/7343070)
Supplement: Supplementary Materials — Fatty acid profiles of Artemia enriched by each experimental emulsions, two experimental emulsions, and postlarvae fed with three experimental treatments. Parameters studied considering their sampling factors, replicate, etc. Figures and tables that are included in this manuscript separately. [file 7343070.f1.zip › Artemia_Postlarvae_Hepatopancrean_Hperimeter4.pdf]

Hepatopancreas perimeter of the postlarvae fed with different Artemia enrichment

enrichment	tank	perh
PA	1	1913,988
PA	1	1983,361
PA	1	2187,121
PA	1	1964,923
PA	1	2021,217
PA	4	2318,396
PA	4	1881,099
PA	4	1776,312
PA	4	1723,809
PA	4	1732,66
PA	7	2275,712
PA	7	1769,038
PA	7	2390,349
PA	7	1411,019
PA	7	2059,768
PB	2	2068,819
PB	2	1637,994
PB	2	1847,283
PB	2	1899,71
PB	2	1666,458
PB	5	1990,732
PB	5	1700,594
PB	5	1715,62
PB	5	1656,265
PB	5	1967,822
PB	8	1862,413
PB	8	1758,5
PB	8	2255,231
PB	8	2671,561
PB	8	2332,99
NE	3	1422,567
NE	3	1352,775
NE	3	2288,217
NE	3	1733,728
NE	3	1852,447
NE	6	1622,697
NE	6	1107,872
NE	6	2358,51
NE	6	1609,244
NE	6	1543,116
NE	9	1662,908
NE	9	1601,803
NE	9	1821,173
NE	9	1381,41
NE	9	1623,264
